# Immediate and Delayed Post Space Preparations in Endodontically Treated Teeth: A Scoping Review

**DOI:** 10.1186/s12903-022-02564-w

**Published:** 2022-12-21

**Authors:** Sadaf Mahmoudi, Pedram Iranmanesh, Saber Khazaei, Maryam Zare Jahromi

**Affiliations:** 1 Department of Endodontics, School of Dentistry, Isfahan (Khorasgan) Branch, Islamic Azad University, Isfahan, Iran; 2 Dental Research Center and Department of Endodontics, Dental Research Institute, School of Dentistry, Isfahan University of Medical Sciences, Isfahan, Iran; 3 Department of Endodontics, School of Dentistry, Kermanshah University of Medical Sciences, Kermanshah, Iran; 4 Department of Endodontics, School of Dentistry, Isfahan (Khorasgan) Branch, Islamic Azad University, Isfahan, Iran

**Keywords:** Post-space preparation, Immediate, Delayed, Leakage

## Abstract

**Background:**

The aim of the present review was to identify the effect of the post-space preparation (PSP) timing (immediate or delayed) on endodontically treated teeth.

**Methods:**

All studies in any language that compared the effect of immediate versus delayed PSP on the outcomes of an endodontically treated tooth were searched in PubMed, Scopus, Web of Science, Cochrane, ProQuest, WorldCat, and Google Scholar databases by two independent researchers until February 12, 2022. Studies assessing merely the immediate or delayed PSP were excluded. A descriptive analysis was performed to evaluate the study design and the characteristics of the included studies.

**Results:**

The initial search yielded 2612 records, of which 68 were included. Except for one randomized controlled trial, all were in vitro. The evaluated variables were apical/coronal microleakage (n = 48/n = 1), post-bond strength (n = 8), bacterial infiltration (n = 7), presence of voids (n = 3), apical extrusion and residual of filling material (n = 1), and post-operative pain (n = 1). The number of publications had steady growth per year and fast growth per decade. "Post-space preparation" was the most popular keywords.

**Conclusions:**

The present review highlights the need for further investigations due to the various design of studies, controversial results, and an inadequate number of randomized controlled trials.

**Trial registration:**

Retrospectively registered. Open Science (10.17605/OSF.IO/2YTM6).

**Supplementary Information:**

The online version contains supplementary material available at 10.1186/s12903-022-02564-w.

## Introduction

Endodontically treated teeth have undergone extensive loss of coronal structure and lack an adequate support for a permanent restoration. In these cases, additional retention from the root canal may be required [1]. Thus, these teeth may need core retention through intracanal post placement [2, 3]. Despite a paradigm shift in dentistry in the last decades regarding replacing the conventional mechanically retained restorations with modern methods which depend on adhesion, these conventional methods of restoring teeth, have been supported by long-term studies as treatment options for restoring endodontically treated teeth because of their good reliability and predictability [4]. Post-space may be prepared by hot pluggers instantly after pulp space obturation or by rotary instruments after sealer setting, categorized as immediate or delayed, respectively [5–7].

During the post space preparation (PSP), the root canal filling material may be twisted, vibrated, or dislocated, consequently creating voids [8]. Several factors, including apical integrity, the remaining amount of root canal filling materials, the obturation techniques, the gutta-percha removal techniques, sealer type, and preparation time, may affect the integrity of root canal filling materials and cause microleakage and bacterial infiltration [2, 5, 6]. Thus, it is essential not to deteriorate the integrity of the residual filling material to provide an appropriate apical sealing and to avoid re-infection or re-colonization of bacteria [5, 6].

It is recommended that the same clinician who has performed the endodontic treatment perform PSP with a rubber dam to avoid contamination [9]. The immediate PSP allows the prepared root canal to receive the post in a unique session and to, assess the condensation of the remaining gutta-percha, and if necessary, some modifications can be considered. However there are disagreements regarding the time interval between the root canal filling and the PSP [7]. The time of PSP may influence some outcomes in endodontically treated teeth such as void formation [10], displacement of filling material [9], the bond strength of the post [5], and post-operative pain [11]. Nevertheless, there are no consensus on the post-operative pain induced by post-endodontic restorations after single- or two-visit root canal treatments [11].

Owing to controversial reports regarding the effect of the interval between root canal treatment and PSP on the outcome of the endodontically treated tooth, it is necessary to identify the existing reports and evaluate the quality of evidence by performing a comprehensive review. Therefore, based on the heterogeneous set of data, this scoping review was conducted to map the research done in this area and to evaluate the outcomes related to immediate or delayed PSP in endodontically treated teeth.

## Materials and Methods

### Protocol and scope of review

The protocol of this study was based on the framework proposed by Peters et al. [12] according to the Joana Briggs Institute. It is available at https://osf.io/yc9nb/. In addition, this scoping review was reported based on the Preferred Reporting Items for Systematic Reviews and Meta-Analyses Extension for Scoping Reviews (PRISMA-ScR) Checklist (Online Resource 1, Table S1). This review focused on the following evidence-based practice: What is the best time for PSP after endodontic treatment?

### Eligibility criteria

Studies that considered the effect of PSP time on endodontically treated teeth were selected. This included experimental studies (in vivo or in vitro), case reports, case series, clinical studies, or systematic reviews that compared the effects of immediate and delayed PSP on endodontically treated teeth. Literature reviews, book sections, congress papers, commentaries, methodological approaches, opinion or hypothesis articles, and editorial letters were excluded.

### Information source and search strategy

An electronic search without date or language restriction was conducted on Medline (PubMed), Scopus, Web of Science and Cochrane databases from their date of inception to February 12, 2022, using the keywords ((((post OR dowel OR fiber post OR intracanal post OR intraradicular post) AND (space AND prepar*)) AND (time OR timing OR immediate* OR early OR late OR delay*))), where asterisk symbol was used for truncation. Grey literature was also searched through ProQuest, WorldCat, and Google Scholar (first 100 hits). Furthermore, the reference lists of included studies, reviews, and textbooks were searched through manual search (Online Resource 1, Table S2).

### Screening

After excluding the duplicated records, the titles and abstracts of the retrieved studies were independently screened by two authors (S.M and P.I). Then the full texts of studies were read and evaluated for the eligibility criteria independently. In all stages, any disagreements were resolved through discussion with the third author (M.Z.J).

### Data extraction

The data of records that met the eligibility criteria were extracted by two reviewers independently(S.M and P.I). A standard form was designed using Excel software (Office, Microsoft, EUA). The studies were categorized into the experimental and randomized controlled trial (RCT) categories according to their design. The data extracted from the included studies were first authors’ last names, year of publication, sample size, time of preparation, and main outcome. Any disagreement was resolved through discussion with other reviewers(M.Z.J). If data were missing, the co-author was contacted via email.

### Bibliometric analyses

The trend line of published articles per year and decade was calculated. The bibliometric data of included articles were retrieved from Scopus and imported as a CSV file to VOS viewer 1.6.15 software (http://www.vosviewer.com/, Centre for Science and Technology Studies, Leiden University, Leiden, the Netherlands) to identify the frequency of journal, country of origin, organization, and authorship involved in publishing the PSP. The hot topics were illustrated using density visualization of author keywords co-occurrence.

### Qualitative analysis

Data were presented on tabular and bar charts based on the number of articles accordant with immediate or delayed PSP or were unclear.

## Results

### Description of the included records

**Fig. 1:**
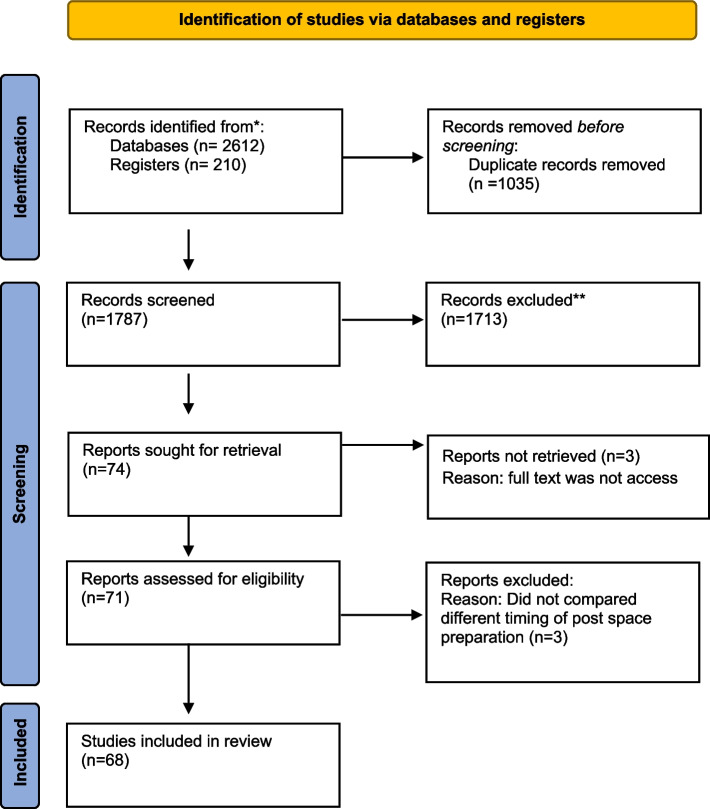
PRISMA flow chart

The initial search yielded 412records from PubMed,911 from Scopus, 588 from Web of Science, 210 from Cochrane library, 385 from ProQuest, 72 from WorldCat, 100 from Google Scholar and 144 from hand search. After the removal of duplicated records (1035), 1713 records were excluded in the initial screening of titles and abstracts. At full-text evaluation stage, 6 records were excluded [13–18]. The reasons for exclusion are described in the Online Resource 1, Table S3. Finally, 68 records published between 1981 and 2022 were included (Figure 1).

### Characteristics of Records

Tables 1, 2, 3, 4, 5, 6 and 7 summarize the main data. According to the study design, one record was RCT, and the rest were experimental studies (67 records). Two studies were performed on bovine teeth, while others were conducted on human teeth.

**Table 1:** Characteristics of the included studies evaluating apical leakage

Author (Year)	Sample size (N)	Groups	System of preparation/Apical size preparation	Filling material / Obturation technique	PSP method	Measurement	Time of immediate PSP	Time of delayed PSP	Outcome
Al-Ashou et al. (2021) [22]	102	G1:Seal Root / G2:AH Plus /G3:GuttaFlow2 SubGroups: I: 24 h/ II: 1W/ GA: 3mm/ GB: 4mm/ GC: 5mm remaining GP	K3 system/ #40, 0.06	GP/ AH Plus, sure seal root canal sealer, GuttaFlow2/ single-cone technique	Peeso reamer #1-4	Penetration depth of MB dye	24 h	7 days	Delay> immediate MIN: sure Seal root sealer 3mm>4mm>5mm
Gujarathi et al. (2021) [26]	60	GA1: AH Plus, imm/ GA2: AH Plus, 1 W/ GB1: sure seal , imm/ GB2: sure seal, 1 W	ProTaper system/ F2	GP/ AH Plus, sure seal root canal sealer / N.r.	Peeso reamer #1-4	depth of dye penetration ,mm, Image Analysis System	Immediately	7 days	Immediate – delay ^*^ sure seal > AH Plus
Tiwari et al. (2021) [25]	66	G1:AH Plus/ G2: Apexit Plus / G3:Endosequence SubGroups: A: imm/ B: delay	ProTaper system/ F3	GP/ AH Plus sealer, Apexit Plus sealer, Bioceramic sealer	Heated plugger Peeso reamer	Penetration depth of MB dye	Immediately	After setting time of the sealers	Delay> immediate Apexit Plus> AH Plus> Bioceramic
Raslan et al. (2020) [1]	20	GA: rotary/NO solvent GB: rotary/ solvent	Mtwo system/ #35	GP/ resin sealer ADSEAL/ WVC	Gates Glidden / solvent (Xylol)	Penetration depth of MB dye in apical third/ stereomicroscope	24 h	7 days	Immediate – delay GA: immediate-delay GB: 24h >one week
Tanompetsanga et al (2020)	60	G1:imm/twisting GP cone G2:1W/hot plugger	Mtwo system/ 50/0.04	GT GP impregnated + BC particles/ BC Sealer/ single-cone technique	Peeso reamer #3	Fluid filtration device	Immediately	7 days	immediate-delay
Jieqi1 et al. (2019)	86	GA1:imm,4mm/ GB1:imm,5mm/ GC1: imm,6mm/ GA2:1W,4mm/ GB2:1W,5mm/ GC2:1W,6mm	ProTaper Universal system/ F2	GP/ AH Plus sealer/ LC	N.r..	glucose microleakage model	Immediately	7 days	delay>immediate IMM: 4mm - 5mm - 6mm Delay: 4mm > 6mm
Lan Y-y et al. (2017) [63]	48	GA1: imm, AH-Plus / GB1:1W, AH-Plus/ GA2:imm, MTA/ GB2: 1W, MTA	N.r./ #30	GP/ AH Plus sealer, MTA/ LC	Peeso reamer #3	Apical dye penetration	Immediately	7 days	immediate-delay AH Plus > MTA
Kim et al. (2017) [23]	50	GI,G8H, G24H, G72H, & G1W	K3 system/40/0.06	GP/ AH Plus sealer/ WVC	Parapost drill, size 1	Linear penetration of the stain; mm	Immediately	8 h, 24 h, 72h & 7days	G1W>GI,G8H, G24H, G72H
Padmanabhan et al. (2017) [6]	130	G1:EndoflasFS,imm/ G2:EndoflasFS, 1W/G3: AH Plus, imm/ G4: AHPlus, 1W/ G5:GuttaFlow, imm/ G6:GuttaFlow, 1W/ G7:MTAFillapex, imm/ G8:MTAFillapex,1W	HyFlex CM/ #40/ 0.04%	GP/sealers: Endoflas FS,AH Plus, GuttaFlow ,MTA Fillapex/ single-cone technique	Gates Glidden 1-4	Linear penetration of the stain; mm	Immediately	7 days	delay>immediate MAX: G2 MIN: G3 G5,G6: immediate-delay
Rui⁃tian et al. (2016)	30	GA: imm/ GB: 2 days/ GC: 7days	ProTaper system/ F3	GP/ AH Plus sealer/ WVC	Fiber post bur	Apical dye penetration	Immediately	48 h, 7 days	Immediate - delay
Nagas et al. (2016) [2]	90	G1: GP+ AH Plus/G2: Resilon + Epiphany/G3: Sealite Ultra/ SubGroups: I: single cone/II: CLC/ III: system B	ProFile system/ 30,0.06%	GP+ AH Plus or Sealite Ultra, Resilon + Epiphany sealer /single cone; CLC, System B	Gates Glidden	Modified fluid transport test; L/min/cmh2o	Immediately	24 h and 7 days	immediate> delay Resilon + Epiphany > Sealite Ultra > AH Plus single cone - CLC - System B
Kala et al. (2015) [30]	70	G1: imm, EndoREZ sealer /G2: 1W, EndoREZ sealer/ G3:imm, RoekoSeal sealer/ G4: 1W, RoekoSeal sealer/	K3 system/ # 30/ 0.06%	GP/ EndoREZ sealer, RoekoSeal sealer/WVC	peeso reamers 1-4	Linear measurement of the dye penetration / stereomicroscope	Immediately	7 days	Immediate - delay EndoREZ> RoekoSeal
Pushpa et al. (2014) [31]	45	G1: imm/ G2: 7days/ G3: 15 days	K-file/ #80	GP/AH Plus/ WVC	heated pluggers Gates Glidden	Apical dye penetration	Immediately	7 days and 15 days	delay>immediate
Dhaded et al. (2013) [7]	80	G1:AH Plus, imm/ G2:AH Plus, 1W/ G3:Resilon/Epiphany, imm/ G4:Resilon/Epiphany, 1W	Protaper universal/ #40	GP/ AH Plus, Resilon + Epiphany/ CLC	peeso reamer	SEM analysis; mm/ stereomicroscope	Immediately	7 days	delay>immediate immediately: AH Plus-Resilon + Epiphany delayed: AH Plus >Resilon/Epiphany
Cheng. Et al (2013)	100	G1: pulp canal sealer /G2: Tubli-Seal EWT /G3: pulp canal sealer EWT/G4: control	K3 system/ #35	GP/ pulp canal sealer, Tubli-Seal EWT, pulp canal sealer EWT/WVC	heated plugger	Apical dye penetration	Immediately	72h and 7 Days	immediate-delay pulp canal sealer > Tubli-Seal EWT, pulp canal sealer EWT
Güngör et al (2013) [34]	44	GA1:GP+AH-Plus imm/ GA2: GP+AH-Plus, 1W/ GE1: EndoREZ, imm/ GE2:EndoREZ, 1W	K-file/ #40	GP/ AH-Plus sealer , EndoREZ /LC	heated pluggers Gates Glidden	computerized fluid filtration device	Immediately	7 days	GE2> GA1- GE1>GA2 EndoREZ > AH-Plus
Pina-Vaz et al. (2013)	40	G1: imm, G2: delay	K-file / N.r.	GP/ Sealapex sealer/ LC	Protaper universal retreatment file	Apical dye penetration	Immediately	8 days	Immediate - delay
Al-Sabawi et al. (2012) [33]	200	G1: CLC, imm/ G2: single-cone, imm/ G3: CLC, 1W/ G4: single-cone, 1W SubGroups: heated pluggers, Gates-Glidden, ProTaper rotary, peeso reamers	ProTaper rotary/ F3	GP/ Tgadseal root canal sealer/ CLC, single-cone technique	heated pluggers/ Gates-Glidden/ ProTaper rotary/ peeso reamers	Apical dye penetration	Immediately	7 days	immediate – delay CLC – single cone technique
Yue et al. (2012)	55	G1: imm/ G2: 2 days/ G3: 1W Sub Groups: Post and core preparation after 15, 30 and 60 minutes of cements mixing	K-file/ #40	GP/ N.r. / LC	Gates Glidden/ Peeso reamer	Apical dye penetration	Immediately	48h, 7 days	7days > 2days> immediate 15 - 30 - 60
dezan junior et al. (2012)	90	Group I (Sealapex, immediately) to Group IX (Topseal, 60 days)	Kerr file/ #60	GP/ Sealapex, Endométhazone, Topseal/ LC	Gates Glidden	Dye infiltration	Immediately	30 days and 60 days	delay>immediate Type of sealer : no statistical differences
Chen & Chang (2011) [37]	100	G1: LC / G2: WVC / G3: injectable thermoplasticized GP / G4: control	K3 system/ 0.4 mm	GP/Sealapex / LC ,WVC , injectable thermoplasticized	heated pluggers	Linear penetration of the stain; mm	Immediately	72h and 7 Days	(Immediate, 3 days) > 7 days
Attam & Talwar (2010) [36]	150	G2: imm, 5mm/ G3: 1W, 5mm/ G4: imm, 3mm/ G5: 1W, 5mm	K3 system/ 45, 0.04%	Epiphany sealer /Resilon system/	Peeso reamers 2,3,4	Fluid transport device	Immediately	7 days	delay>immediate 3mm > 5mm
Paiva et al. (2010) [41]	40	G1: imm, ZOE/ G2: ZOE, delay	N.r.	GP/ ZEO sealer/ LC	ProTaper Universal retreatment files/ H-files	Apical dye penetration	Immediately	8 days	delay>immediate
Aydemir et al. (2009) [8]	64	G1: CLC, Sealapex, 30 days / G2: CLC, Sealapex, 40 min/ G3: CLC, Diaket, 30 days/ G4: CLC, Diaket,40 min/ G5: WVC, Diaket, 30 days/ G6: WVC, Sealapex, 30 days	K-file/ #50	GP/ Sealapex , Diaket /CLC, WVC	Gates Glidden 1-4	Electrical current; micro-siemens	40 min	30 days	immediate-delay Sealapex- Diaket CLC > WVC
Chen Mei et al. (2009) [44]	60	GA1:imm, temporary materials / GA2:imm,fiber post and cores / GB1: 1W,temporary materials / GB2: 1W,fiber post and cores	hand ProTaper instruments/ F3	GP/ AH Plus/ LC	Peeso reamers #2	Apical dye penetration	Immediately	7 days	delay>immediate fiber post and core - temporary materials
Kalra et al (2009) [64]	70	G1: AH-Plus, imm /G2: AH-Plus, 1W/ G3: RC Seal, imm/ G4: RC Seal, 1W	K-file/ #50	GP/ AH-Plus, RC Seal sealer/ E & Q Plus system	Peeso reamers #3	Apical dye penetration	Immediately	7 days	delay>immediate RC Seal > AH-Plus
Ehsani et al. (2009) [39]	76	G1: AH26, imm/ G2: AH26, 1W/ G3: Apatite root sealer , imm,/ G4: Apatite root sealer , 1W	K-file/ #35	GP/ AH26, Apatite root sealer / LC	Peeso reamers #3	Apical dye penetration	Immediately	7 days	delay>immediate AH26 - Apatite root sealer
Yildirim et al. (2009) [43]	51	G1: GP+AH-Plus+LC , imm / G2: GP+AH-Plus+LC , 1 W/ G3: 5 mm MTA	K-files/ #60	GP/ AH-Plus sealer , MTA plug /LC	heated pluggers Gates-Glidden	Computerized fluid filtration method	Immediately	7 days	immediate-delay (GP/ AH-Plus sealer) > MTA
Cobankara et al. (2008) [38]	50	G1: CLC, EndoREZ (A_), imm / G2: CLC, EndoREZ (A+), imm / G3: CLC, EndoREZ (A_), 1 W/ G4: CLC, EndoREZ (A+),1 W	ProFile system/ 0.465 mm	GP/ EndoREZ sealer with or without accelerator / CLC	heated pluggers	Computerized fluid filtration method	Immediately	7 days	delay>immediate (A+) - (A_)
Salim et al. (2008) [42]	40	G1a: imm, heated pluggers/ G1b: imm, peeso reamer/ G2a: 1W, heated pluggers/ G2b: 1W, peeso reamer	hand ProTaper instruments/ F1,F2,F3	GP/ Zinc oxide eugenol sealer/ LC	heated pluggers peeso reamer	Apical dye penetration	Immediately	7 days	delay > immediate MIN: G1a MAX: G2b
Corrêa Pesce et al. (2007) [20]	60	GA1: EndoFill , no post/ GA2: EndoFill, 24 h/ G A3: EndoFill, 72h/ G B1: AH-Plus, no post/ G B2: AH-Plus, 24 h/ G B3: AH-Plus, 72 h	K-file/ #55	GP/ EndoFill or AH-Plus sealer/ CLC	heated pluggers	Apical dye penetration	24 h	72 h	Immediate - delay EndoFill > AH-Plus In all groups: 1mm > 2mm >3 mm from the apex
Bodrumlu et al. (2007) [47]	72	G1:AH Plus, imm/ G2:AH Plus, 1W/ G3:Resilon/Epiphany, imm/ G4:Resilon/Epiphany, 1 W	K-file/ #40	GP/ AH-Plus or Resilon + Epiphany /LC	Gates-Glidden 3-4	Fluid transport device	Immediately	7 days	GP/AH Plus: delay > immediate Epiphany/Resilon: Immediate > delay Immediate: (AH-Plus) –(Resilon Epiphany) Delay:(AH-Plus) > (Epiphany/Resilon)
Shahi et al. (2007) [51]	96	GA: peeso reamer, imm/ GB: peeso reamer, 1W/ GC: heated pluggers, imm/ GD: heated pluggers, 1W	K-file/ #35	GP/ AH26 sealer/ LC	heated pluggers /peeso reamer	Apical dye penetration	Immediately	7 days	Immediate - delay MIN: GC MAX: GB heated pluggers - peeso reamer
Sadeghi et al. (2007) [50]	74	G1: LC, imm/G2: LC, 1W/ G3: WVC, imm /G4: WVC, 1W	K-file/ #40	GP/ Tubliseal sealer/ LC, WVC	heated pluggers	Apical dye penetration	Immediately	7 days	delay>immediate WVC > LC
Javidi et al. (2007) [49]	76	G1:AH 26, imm/ G2:AH 26, 1W/ G3:Apexit Plus , imm/ G4:Apexit Plus , 1W	K-file/ #45	GP/ AH26 sealer, Apexit Plus / LC	heated pluggers	Apical dye penetration	Immediately	7 days	delay>immediate G4> G1 AH26 -Apexit Plus
Solano et al. (2005) [52]	46	G1:AH Plus, imm/ G2:AH Plus, 1W	K3 system/ #30 .06	GP/ AH-Plus sealer/ WVC	heated pluggers / Gates-Glidden	Apical dye penetration	Immediately	7 days	delay>immediate
Rastegar et al. (2004) [48]	76	G1: AH26, imm/ G2: AH26, 7 days/ G3: Roth sealer, imm/ G4: Roth sealer, 14 days	K-file/ #45	GP/ AH26, Roth sealer/ LC	peeso reamer	Apical dye penetration	Immediately	7 days and 14 days	delay>immediate MIN: G1 MAX:G4 AH26 - Roth sealer
Ashraf et al. (2003) [46]	96	G1:imm, AH26/ G2: delay, AH26/ G3: imm, Apexit/ G4: delay, Apexit	K-file/ #40	GP/ AH26, Apexit/ LC	peeso reamer #2,3	Apical dye penetration	Immediately	7 days	Immediate - delay AH26- Apexit
Abramovitz et al. (2000) [45]	53	GA:imm/ heated pluggers GB: 1W/Gates-Glidden 3-4	K-file/ #35	GP/ AH-Plus sealer/ LC	heated pluggers / Gates-Glidden 3-4	Pressure-driven system; cpm, Leakage without Pressure, and Leakage under Pressure	Immediately	7 days	immediate-delay heated pluggers- drills
Fan et al. (1999) [55]	90	G1: AH26, imm / G2:AH26, 1W/ G3: Pulp Canal Sealer ,imm/ G4: Pulp Canal Sealer, 1W	K-file/ #50	GP/ AH26 or Pulp Canal Sealer / LC	Gates-Glidden #90	Fluid transport device	Immediately	7 days	delay>immediate
Karapanou et al. (1996) [56]	80	G1: imm, Roth 801 sealer/ G2:1W, Roth 801 sealer / G3:imm, AH26 / G4:1W, AH26	N.r.	GP/ Roth 801 sealer, AH26/ CLC	Gates-Glidden	Linear penetration of the stain; mm	Immediately	7 days	Roth 801 sealer: delay>immediate AH-26 sealer: immediate-delay
Rybick & Zillich (1994) [60]	60	G1: imm, Thermafil / G2:delay, Thermafil / G3:noPSP, Thermafil / G4: no PSP, CLC	K-file/ #35-70	Thermafil, ThermaSeal epoxy resin-based sealer /CLC	Thermafil Prepi burs	Volumetric analysis; absorbance	Immediately	72 h	Immediate - delay
Saunders et al. (1993) [61]	72	G1: imm/ G2: 1 W	Flex 0 Files/ #40-50	Thermafil/ Sealapex	Peeso reamer #110	Apical dye penetration ; score	Immediately	7 days	Immediate - delay
Moon et al. (1988) [58]	76	GA1: imm, thermatic condensation/ GA2: delay, LC/ GB1: imm, thermatic condensation/ GB2: delay, LC	H-file/ #25	GP/ AH26 sealer/ thermatic condensation, LC	heated pluggers/ Gates-Glidden/ Peeso reamers	Apical dye penetration	Immediately	7 days	Immediate - delay LC > Thermatic condensation
Madison & Zakariasen (1984) [57]	80	G1: imm, solvent/ G2:imm, heat/ G3:imm, drill / G4: 48h, solvent/ G5:48h, heat /G6: 48h, drill/	K-file/ #40	GP/ Roth's root canal cement/ LC	Peeso reamers/ heated pluggers/ chloroform & files	(Linear penetration of the stain; mm) and (*Volumetric analysis; μl)	Immediately	48h	immediate-delay solvent - heat – rotary instrument
Portell et al. (1982) [59]	47	G1: imm, 3mm/ G2: imm, 7mm/ G3: 2 W, 3mm/ G4: 2 W, 7 mm	K-file/ #50	GP/ non staining root canal cement/ CLC	heated pluggers	(Autoradiographic investigation; mm) (Analysis of the incidence; (number teeth with leakage) & degree of leakage: mm)	Immediately	2 week	delay > immediate 3mm > 7mm (At all distances from W.L)
Dickey et al. (1982) [54]	60	GA1: Peeso reamers, 1 W/ GA2: Peeso reamers, imm/ GB1: solvent, 1 W/ GB2: solvent, imm	K-file/ #55	GP/ Grossman's sealer/ LC	Peeso reamers/ chloroform & files	Autoradiographic investigation; mm	Immediately	7 days	Immediate > delay solvent - rotary instrument
Bourgeois & Lemon (1981) [26]	44	GA: delay /GB: imm	N.r.	GP/ AH26 or Grossman's sealer / LC	heated pluggers	Autoradiographic investigation; mm	Immediately	7 days	Immediate –delay AH26 > Grossman

(A+)/(A_); with or without accelerator, CLC; cold lateral compaction, G; group, GP; gutta-percha, h; hours, imm; Immediate, L; lateral condensation, MB; methylene blue, mm; millimeter, N.r.; not reported, PSP; post space preparation, W; week, WL; working length, WVC; warm vertical compaction, ZOE; zinc oxide eugenol sealer, - ; similar outcome.

**Table 2:** Characteristics of the included studies evaluating coronal microleakage

Author (year)	Sample size (N)	Groups	System of preparation/Apical size preparation	Obturation technique	PSP method	Measurement	Time of immediate PSP	Time of delayed PSP	Outcome
Lan Y-y et al., (2016) [63]	48	Based on the sealer: AH-Plus, MTA	N.r./ #30	Lateral condensation: GP/ AHPlus or MTA	Peeso reamer #3	Dye penetration	Immediately	7 days	AH Plus: delay MTA: -

PSP; post space preparation, GP: gutta-percha, N.r.; not reported, - ; similar outcome

**Table 3:** Characteristics of the included studies evaluating bacterial infiltration

Author (Year)	Sample size (N)	Groups	Rotary system/ Apical size	Filling material/ Obturation technique	Bacteria type	PSP technique	Measurement	Time of immediate PSP	Time of delayed PSP	Outcome
Sharma et al. (2020) [68]	32	GA: CLC, imm/ GB:MTA plug,imm/ GC:4mm MTA plug, delay	K-file/ #40	GP/ MTA plug	N.r.	N.r.	bacterial leakage test	Immediately	N.r.	Immediate – delay GA> GB – GC GP > MTA plug
Reyhani et al. (2015) [69]	76	G1: AH Plus, imm/ G2: AH Plus, 1W/ G3: MTA Fillapex, imm/ G4: MTA Fillapex, 1W	Race rotary files/ #25, 6%	GP/ MTA Fillapex , AH Plus sealer/ LC	E.F/ 90 days	Peeso reamer #3	bacterial microleakage system; number of samples with leakage during study	Immediately	7 days	Immediate - delay MTA Fillapex - AH Plus
Nikhil et al. (2011) [66]	90	G1ax: AH 26, imm, 5mm GP/ G1ay: AH 26, imm ,4mm GP+1mm GIC/ G1bx: AH 26, 1W, 5mm GP/ G1by: AH 26, 1W, 4mm GP+1mm GIC/ G2ax: Resilon+Epiphany, imm, 5mm resilon/ G2ay: Resilon+Epiphany, imm, 1mm GIC/ G2bx: Resilon+Epiphany, 1W, 5mm resilon/ G2by: Resilon+Epiphany, 1W, 4mm GP+1mm GIC	K-file/ #40	GP/AH26 , Resilon/Epiphan/ LC	Staphylococcus species/ 90 days	heated pluggers	bacterial microleakage test	Immediately	7 days	Delay > immediate
Jalalzadeh et al. (2010) [67]	86	GA: imm, AH26/ GB: imm, ZOE/ GC: 1W, AH26/ GD: 1W, ZOE	K-file/ #35	GP/ AH26, ZOE sealer/ LC	Staphylococcus epidermidis/ 70 days	Peeso reamer #3	bacterial microleakage test	Immediately	7 days	Immediate - delay ZOE: Immediate - delay AH26: Delay > immediate ZOE – AH26
Zmener et al. (2010) [19]	48	G1: EndoREZ (A_), 22min / G2: EndoREZ (A+), 8 min / G3: EndoREZ (A_), 1 W / G4: EndoREZ (A+),1 W	K-file/ #40	GP/ EndoREZ sealer with or without accelerator / LC	E.F/ 60 days	Gates Glidden #2-5/ Para Post System drill #5, 5.5, 6	Dual-chamber microbial leakage model; % teeth without coronal bacterial leakage	22 min, 8 min	7 days	Delay > immediate (A+) - (A_)
Lyons et al. (2009) [71]	80	G1: imm/ G2: delay/ G3: imm/ G4: delay	EndoSequence/ #50 .06	Resilon + Epiphany sealer / WVC-System B- Obtura II	S.M/ 3, 7, 10, 14 up to maximum 28 days	System B + Gates Glidden	Dual-chamber microbial leakage model; % teeth with bacterial leakage	Immediately	5 days	Immediate - delay all groups showed leakage after 14 days: failure of the Resilon/Epiphany to create a true ‘monoblock’
Grecca et al. (2009) [70]	66	G1: imm, burs/ G2: 1W, burs/ G3: imm, heated pluggers / G4: 1W, heated pluggers/ G5: imm, solvent/ G6: 1W, solvent	K-file/ #50	GP/ AH Plus sealer/ Tagger’s hybrid technique	E.F/ 90 days	LA Axxess Bur/ heated pluggers /solvent xylol	Dual-chamber device; number of teeth with bacterial leakage	Immediately	7 days	Immediate - delay Bur - heated pluggers - solvent

(A+)/(A_); with or without accelerator, *CLC* cold lateral compaction, *G* group, *GP* gutta-percha, *imm* Immediate, *L* lateral condensation, *mm* millimeter, *MTA* mineral trioxide aggregate, *N.r*. not reported, *PSP* post space preparation, *W* week, *WVC* warm vertical compaction, *ZOE* zinc oxide eugenol sealer, - ; similar outcome.

**Table 4:** Characteristics of the included studies evaluating voids

Author (Year)	Sample size (N)	Groups	System of preparation/ Apical size	Filling material/ Obturation technique	PSP method	Measurement	Time of immediate PSP	Time of delayed PSP	Outcome
Kataia1 et al. (2020)	56	GA1: penetrating drill/ GA2: heated plugger & EXACTO bur SubGroups: single cone, System-B Minor SubGroups: imm, 1W	ProTaper Universal System/ F5	GP/AH Plus sealer /single cone, System-B	penetrating drill, heated plugger & EXACTO bur	By micro-CT scanner; % voids	Immediately	7 days	Delay > Immediate single cone > System-B penetrating drill > heated plugger & EXACTO bur
Long et al (2019) [5]	40	GA: single cone, imm/ GB: CWC, imm/ GC: single cone , 1W/ GD: CWC, 1W	M3 Rotary system/ 30.04	GP/ iRoot SP sealer/ single cone or CWC/	heated plugger	By micro-CT imaging; % volume	Immediately	7 days	immediate-delay CWC - single cone
Dhaded et al. (2013) [72]	80	G1:AH Plus, imm/ G2:AH Plus, 1W/ G3:Resilon/Epiphany, imm/ G4:Resilon/Epiphany, 1W	Protaper universal/ #40	GP+ AH Plus, Resilon + Epiphany/ CLC	peeso reamer	SEM analysis; mm/ stereomicroscope	Immediately	7 days	Delay > immediate immediately: AH Plus-Resilon + Epiphany delayed: AH Plus >Resilon/Epiphany

*CLC* cold lateral compaction, *CWC* continuous wave of condensation, *G* groups, *GP* gutta percha, *imm* Immediate, *PSP* post space preparation, *W* week, - ; similar outcome.

**Table 5:** Characteristics of the included studies evaluating bond strength of the posts

Author (Year)	Sample size (N)	Groups	System of preparation/ Apical size/ Irrigation Protocol	Obturation material/ Technique	PSP method/ Dentin adhesion/ Luting agent	Measurement	Time of immediate PSP	Time of delayed PSP	Outcome
Long et al (2019) [5]	40	GA: single cone, imm/ GB: CWC, imm/ GC: single cone , 1W/ GD: CWC, 1W	M3 Rotary system/ 30.04/ 2.5% NaOCl, 17% EDTA, 1% NaOCl	GP/ iRoot SP sealer/ single cone or CWC	heated plugger/ ScotchbondTM Universal Adhesive/ RelyX	Push-out test in fiber post; mpa	Immediately	7 days	immediate-delay^*^ CWC - single cone
Vilas-Boas et al (2017) [73]	84	GA1: imm, Endofill/ GB1: imm, BC Sealer/ GC1: imm, AH Plus/ GA2: 1W, Endofill/ G B2: 1W, BC Sealer / GC2: 1W, AH Plus	Reciproc/ R40/ 2.5% NaOCl, 17%EDTA	GP/ Endofill Endosequence BC Sealer, AH Plus / thermo filling with McSpadden compactor	Largo burs #3/ ScotchbondMulti Purpose / RelyX	Micropush-out bond strength of the fiber post; mpa	Immediately	7 days	Immediate > delay AH Plus sealer >Endofill, Endosequence BC
Aleisa, et al (2016) [21]	72	G1: 24 h G2: 2 W	Protaper system / F2/ 5.25% NaOCl	GP/ Endofil sealer / LC	Peeso reamer #5/ ParaBond adhesive A/B/ Variolink II, RelyX, ParaCoreColtèene/	pull-out test of fiber post (parapost)	24 h	14 days	Immediate > delay 24h: RelyX>Variolink II, ParaCore 2 W: RelyX>Variolink II
Machado et al. (2015) [77]	12	G1: imm/ G2: 21 days	Protaper universal system/ N.r./ 2.5% NaOCl	GP/ AH Plus / WVC	Largo drills 3-5/ self-adhesive Resin cement Rely-X	Push-out test in cylindrical fiber post; mpa (Cervical ,medium and apical third)	Immediately	21 days	Delay > Immediate Imm: Cervical - medium -apical third Delay: apical > Cervical, medium third
Dias et al. (2009) [62]	60	GI: imm/ GII: 72 h/ GIII: 4 months	K-file/ #45/ 1% NaOCl	GP/ Endofill/ LC	Largo drills 1-3/ Kuraray adhesive system / zinc phosphate cement, Panavia F	Push out test in the cylindrical SS post	Immediately	72 h and120 days	immediate-delay zinc phosphate >Panavia F
Vano et al. (2008) [74]	68	G1: imm/ G2: 24 h/ G3: 1 W	K-files (#08-10-15), M-two (#10-15-20-25), Profiles .06 (#30-35-40)/ 5.25% NaOCl	GP/ AH Plus sealer/ CWC	heated plugger / Monobond-S	Push out test in post; mpa ( ENA post , DT Light Post and FRC Postec)	Immediately	24 h and 7 days	Delay > Immediate FRC Postec, DT Light Post > ENA post
Vano et al. (2006) [75]	60	G1: imm/ G2: 24h/ G3:1W	K-files (#08-10-15), M-two (#10-15-20-25), Profiles .06 (#30-35-40)**/** 5.25% NaOCl	GP/ Pulp Canal sealer/ CWC	low-speed post drills	(Push out test in the post; mpa) FRC Postec , ENA Post and DT Light Post	Immediately	24 h and 7 days	Delay > Immediate FRC Postec, DT Light Post > ENA post
Boone et al. (2001) [76]	120	G1: imm, Roth’s 801, O/S/ G2: imm, AH26, O/S/ G3: imm, , Roth’s 801, S/O/ G4: imm, AH26, S/O/ G5: 1W, Roth’s 801, O/S/ G6: 1W, AH26, O/S/ G7:1W, Roth’s 801, S/O/ G8: 1W, AH26, S/O	ProFile system /50, .04/ 5.25% NaOCl, 17% EDTA	GP/ Roth’s 801, AH26/ LC	Touch ’n Heat/ Panavia 21	(Push out test in the #6 parallel-sided stainless-steel Parapost XP post)	Immediately	7 days	immediate-delay Roth’s 801 - AH26 O/S > S/O

*CWC* continuous wave of condensation G; group, *GP* gutta-percha, *h* hours, *imm* Immediate, *L* lateral condensation, *N.r.* not reported, *O/S- S/O* obturated before post space prepared - post space prepared before obturation, *PSP* post space preparation, *W* week, *WVC* warm vertical compaction, - ; similar outcome

**Table 6:** Characteristics of the included studies evaluating apical displacement and residual filling material

Author (Year)	Sample size (N)	Groups	System of preparation / Apical size	Filling material/ Obturation technique	PSP method	Measurement	Time of immediate PSP	Time of delayed PSP	Outcome of residual filling	Outcome of apical displacement (extrusion)
Rosatto et al (2020) [9]	20	G1: imm, drill/ G2: imm, thermo/ G3: 2 W, drill/ G4: 2 W, thermo	K-file/ #30	GP/ AH Plus sealer /single cone	Peeso reamers 2-3 , thermo using M and FM tips – Odous Touch	Micro-CT Analysis; volume (mm3) in cervical, middle and apical thirds	30 min	14 days	Both Methods: Delay > Immediate Cervical, Middle: drill - thermo Apical: thermo > drill Imm: cervical- middle -apical All Other Combinations: middle ,apical > cervical	immediate-delay thermo: imm, delay drill: imm

G; group, GP; gutta-percha, h; hours, imm; Immediate, PSP; post space preparation, W; week, - ; similar outcome

**Table 7:** The clinical trial study evaluating post operative pain after PSP

Author (Year)	Sample size (N)	Prescribed analgesics	System of preparation/ Apical size	Filling material/ Obturation technique	PSP method	Postoperative pain evaluation	Time of immediate PSP	Time of delayed PSP	utcome
Eyuboglu et al. (2020) [11]	100	(naproxen sodium, 550 mg) at the end of the first appointment	2Shape rotary system/ TS2 (#25/.06) or 2Shape F35 (#35/.06)	GP /calcium silicate-based root canal sealer / Single cone	fiber post drills	An electronic pain rating scale program + (VAS) in 1, 2, 3 days and 1 week after the first visit	first-visit: immediately	second-visit :at least 1 W	1-visit > 2-visits

*G*, group, *GP* gutta-percha, *imm* Immediate, *PSP* post space preparation, *VAS* visual analogue scale, *W* week, - ; similar outcome

### In Vitro Studies

For the in vitro category, 67 studies were included (Tables 1, 2, 3, 4, 5 and 6). Regarding the timing of PSP, the immediate preparation times were 8 min and 22 min [19], 30 min [9], 40 min [8] in one study each, and 24 hours in four studies [1, 20–22]. The exact time of immediate PSP was not reported in other included studies [2, 6, 7, 10, 23–61]. There was a great range regarding the exact time of delayed PSP. For the delayed preparation group, the included studies considered a wide range from4 months [62] to 48 h or 72 h [20, 23, 28, 29, 35, 37, 57, 60, 62]. One study evaluated the effect of four delayed preparation times, including 8 h, 24 h, 72 h, and 7 days [23].

**Fig. 2:**
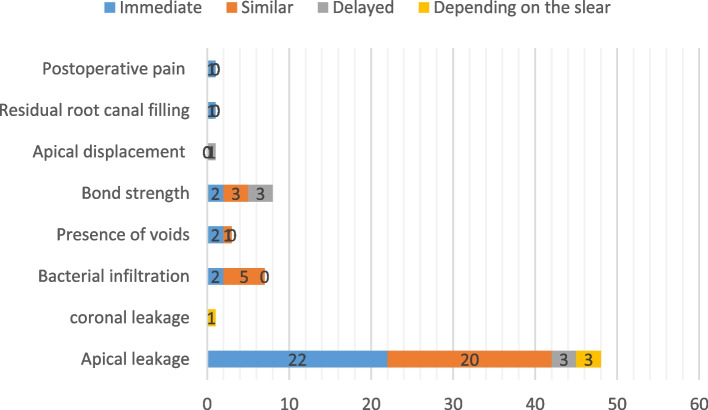
The summery of superiority of delay vs immediately post space preparation according the included studies

In experimental studies, the effects of timing of PSP on 6 variables, including graphical microleakage (*n*=48), coronal microleakage (*n*=1), bacterial infiltration (*n*=7), presence of void (*n*=3), bond strength (*n*=8), and apical displacement and residual root canal filling (*n*=1), were evaluated (Tables 1, 2, 3, 4, 5 and 6 and Fig. 2).

#### Apical Microleakage

Apical microleakage was assessed in 48 in vitro studies [1, 2, 6–8, 20, 22–61, 63, 64] using different methods such as autoradiographic investigation [53, 54, 59], linear dye penetration [1, 6, 20, 22, 23, 25, 26, 28–33, 35, 37, 39–42, 44, 46, 48–52, 56–58, 61, 63, 64], glucose microleakage model [27], fluid transport device [2, 24, 36, 47, 55], volumetric analysis [60], computerized fluid filtration method [34, 38, 43], electrical curren t[8], pressure-driven system [45], and scanning electron microscopy (SEM) [7]. Different outcomes were reported in this regard. While most studies showed the benefit of immediate PSP [6, 7, 22, 23, 25, 27, 31, 35, 36, 38–42, 44, 48–50, 52, 55, 59, 64], others showed no superiority of either timing [1, 8, 20, 24, 26, 28–30, 32, 33, 43, 45, 46, 51, 53, 57, 58, 60, 61, 63], and a few showed lower apical microleakage after delayed PSP [2, 37, 54]. In three studies based on the type of sealers used, different results were achieved, which will be discussed further [34, 47, 56].

#### Coronal Microleakage

A study examined coronal microleakage using different filling materials [65] and showed the length of coronal microleakage depended on the sealer and timing of the PSP. When mineral trioxide aggregate (MTA) paste was used as a filling material, the timing of PSP did not affect coronal microleakage. However, when AH plus paste was used, immediate PSP resulted in lower coronal microleakage (Table 2).

#### Bacterial Infiltration

Seven *invitro* studies evaluated bacterial infiltration [19, 66–71], three used *Enterococcus faecalis* [19, 69, 70], two used *Staphylococcus* species [66, 67], and one used *Streptococcus mutans* as the bacterial marker [71] for the preparation of the infected specimens. The microbial microleakage model in three studies consisted of the dual chamber test [70, 71] or a slight modification of it [19], and four studies used the bacterial microleakage system, which provides qualitative rather than quantitative results [66–69]. All the studies considered extracted human single-rooted teeth. Overall, they found no significant difference in the bacterial microleakage between immediate and delayed PSP groups, except two studies [19, 66], which reported lower bacterial infiltration was reported in the immediate PSP group.

#### Presence of Voids

Three studies evaluated the presence of voids; one with SEM [72] and the two other with micro-computed tomography (micro-CT) imaging [5, 10]. The micro-CT imaging showed no significant differences between the immediate or delayed PSP [5] based on the measurement of the volume of voids. However, another micro-CT imaging analysis reported the lowest mean value of voids in the immediate PSP group [10], the same results as those of another SEM analysis [72].

#### Bond Strength

Considering the bond strength, all eight studies analyzed adhesive resistance by the push-out test of the post after cementation. They sectioned the specimens perpendicularly to the long axis of the root, and a cylindrical plunger was applied in the apical-coronal direction loading until the filling material or the post was dislodged from the slice. Teeth that were used were human single-rooted teeth in 6 studies [21, 62, 73–76], human premolar teeth with double root canals in one study [5], and bovine teeth in one study [77]. Various techniques and materials were used for post cementation. Experimental procedures were done on fiber posts in all studies, whereas stainless-steel posts were used in two studies [62, 76]. Sodium hypochlorite (NaOCl) [5, 21], NaOCl and EDTA 17% [76], and distilled water [73, 78] were among the solutions used for post space irrigation. Moreover, in a study by Vano et al. [75], an additional SEM analysis was done to assess the cleanliness of the post-space qualitatively. In general, the outcomes of the studies were heterogeneous. Three studies indicated no significant differences [5, 62, 76], while three [74, 75, 77] and two studies [21, 73] reported higher bond strength in the delayed and immediate preparation groups, respectively.

#### Apical displacement and residual root canal filling

A study analyzed apical displacement and residual root canal filling [9]. Bovine teeth were scanned by micro-CT before and after PSP. No significant difference was reported for the displacement of the filling material in the apical direction (extrusion). However, regarding residual root filling, immediate PSP, either with thermo or drill methods, resulted in lower residues in the root canal. In general, PSP with a drill resulted in more homogeneous root canal preparation, mainly when performed immediately.

### Clinical studies

An electronic pain rating scale program with a vertical visual analog scale (VAS) was used after the first visit for both groups to evaluate post-operative pain by an RCT (Table 7) [11]. Results presented no post-operative pain in patients with delayed post cementation, in contrast, there was significantly higher post-operative pain in immediate post installation on days 1, 2 and 3 and 1 week after the first visit.

### Bibliometric Analysis

**Fig. 3:**
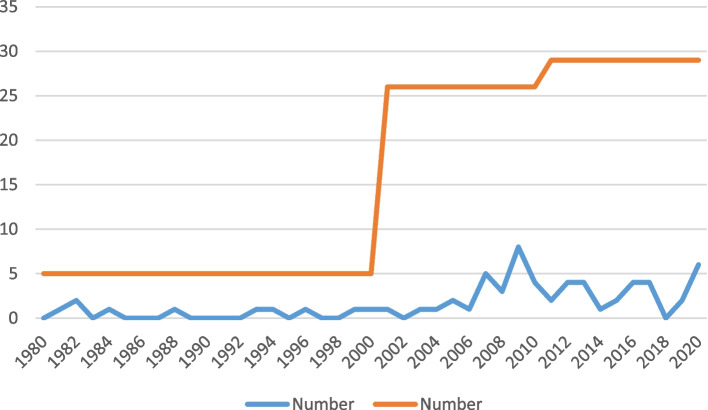
The trend of published studies regarding immediate and delayed PSP comparison over the past 40 years per year (blue line) and decade (red line)

**Fig. 4:**
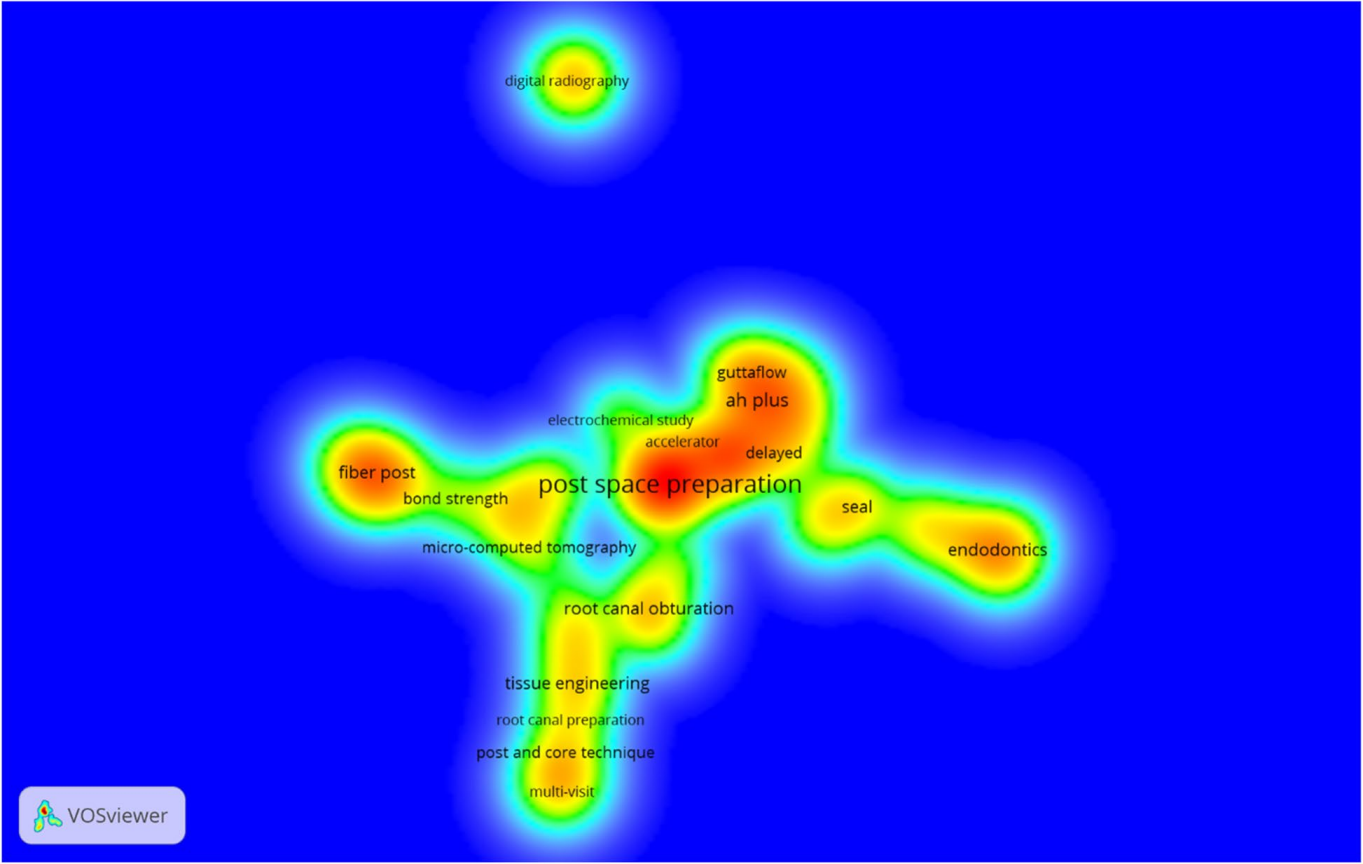
Author hot topics among 67 included articles using density visualization. Red–yellow–green–blue color scheme was used; red corresponds to the highest and blue corresponds to the lowest density of keyword co-occurrence. The distance-based approach was used; smaller the distance between two item means the higher relatedness

Linear trend line analysis showed that the number of publications on PSP in endodontology had a steady growth per year but had a fast growth per decade (Fig. 3). Totally, 44 journals contributed to publishing articles on PSP, of which “Journal of Endodontics” with 11 published articles, acquired the top rank (Online Resource 1, Figure S1). Among19 countries that contributed to the research and publications in the field of immediate and delayed PSP, India had the highest number of publications (n = 11), followed by the USA, China, and Iran (n = 8 for each) (Online Resource 1, Figure S2). Furthermore, the U.S.A. with, 5 links had the highest international collaboration (Online Resource 1, Figure S3). At the author level, 175 authors within 37 clusters from 88 organizations contributed to the publishing of articles on PSP (Online Resource 1, Figure S4). Density visualization indicated that “post-space preparation”, “fiber post", and “AH plus” were the most popular keywords used by authors (Fig. 4).

## Discussion

This scoping review provides information about the publishing metrics of PSP. Articles with a steady growth evaluated the influence of different time intervals of PSP on various aspects of an endodontically treated tooth over four decades. It should be noted that there was no well-designed randomized controlled trial on this subject. Furthermore, the present scoping review categorized the effect of the timing of PSP on the apical sealing of the remaining root canal filling material, bacterial infiltration, presence of voids, post-bond strength, apical extrusion, residual filling material, and post-operative pain in endodontically treated teeth. Overall immediate PSP would be the safest clinical choice regarding most of these parameters. Other than post-operative pain, there were lower apical microleakage and lower residues into the root canal after immediate PSP, and no difference was found regarding bacterial microleakage and displacement of filling materials. However, further studies are required to explore some parameters such as the presence of voids and post-bond strength.

The data regarding apical microleakage were very heterogeneous, and most of the in vitro studies indicated lower penetration of dyes or fluids into the immediate PSP some suggesting no difference in bacterial infiltration. However, a recent meta-analysis with strict eligibility criteria, which included 3 studies, reported higher apical microleakage in one week delayed PSP [79]. Moreover, a systematic review reported all techniques used for evaluating apical microleakage were helpful if the study had a large sample size and a proper control group [80]. In addition to these factors, variations in sealer type and root filling materials, PSP methods, and the amount of the remaining root filling material may be other reasons for this difference [2, 47, 79]. Regarding the different properties of the sealers, a lower apical microleakage was shown in the immediate PSP using the AH plus compared with delayed PSP and zinc oxide-eugenol-based seale r[6, 20, 48, 53, 55, 56]. For AH plus, higher adhesion to dentin and expansion capacity may significantly reduce the apical microleakage [5, 45, 55]. Different PSP methods and the amount of remaining root filling materials may affect the outcome of apical microleakage. For instance, a heated endodontic plugger for immediate PSP may remove the gutta-percha without disturbing the apical seal and eliminate the possibility of inadvertent damage to dentin [81]. In any case, PSP should allow a minimum length of 3-5 mm gutta-percha in the apical third to preserve the seal [81]. In two studies comparing the effect of the length of the remaining apical filling material on apical microleakage, the more was the remaining apical filling material, the lower was the apical microleakage [22, 27]. In conclusion, many factors may affect the outcome of apical microleakage, so more studies are required to be conducted in this regard.

Among three studies that evaluated the presence of void and adaptation of filling materials, Dhaded et al. [72] and Kataia et al. [10] reported better adaptation in the immediate PSP group. On the other hand, no difference was reported by Long et al. [5] which might be due to different obturation techniques and sealers. For instance, the single-cone technique provides void-free obturation and improves the apical sealing along with a minimal sealer thickness [2]. The delayed PSP may cause the fracture of the bonding of the sealer (AH Plus, Resilon / Epiphany) to dentin when the sealer is set [10]. Still, considering the excellent properties of calcium silicate-based sealers, the bonding integrity is maintained [4]. In general, due to limited studies, more studies are suggested to be performed in this regard.

Bond strength was evaluated by the push-out test in the included studies [5, 21, 62, 73–76, 78] with controversial results regarding the timing of PSP. The adhesive system, surface treatment, and cement curing methods that guaranteed efficient adhesion of the post to the root dentin [82] were different and might be the reason for controversy. Post-space surface treatment such as the use of ethanol or sodium hypochlorite removes the smear layer efficiently and increases the infiltration depth of the adhesive and types of cement into the dentinal tubules [83, 84]. Regarding the effect of the cement curing methods on bond strength, dual or chemically activated adhesive resin cements are the most appropriate for the cementation of fiberglass posts since, with a longer working time, they allow better control of the post adaptation [82]. The use of photoactivated cement, because of limited light transmission to the apical third, results in a low degree of conversion of resin monomers, thereby compromising the integrity of the adhesive interface [82]. Future studies are required to investigate the influence of these factors and an appropriate time of PSP that guarantee effective bond strength.

Apical displacement and residual root filling after PSP were analyzed through micro-CT analysis in one study [9]. The micro-CT provides high-resolution images and allows for quantitative analyses. While no difference was reported regarding apical displacement of the filling material, a lower percentage of residual filling material was reported for immediate PSP, which is probably related to the technical difficulty of root filling material removal over time.

A randomized controlled trial evaluated the post-operative pain of PSP and showed the vibrations generated during post-installation by a low-speed handpiece with dedicated fiber post drills from the post kit in the same visit of root canal filling might increase the post-operative pain. Therefore, the cumulative effect of root canal treatment and post installation in the first visit might increase the intensity of pain [11]. Although delayed PSP was recommended, more studies are necessary to thoroughly investigate the effects of the timing of PSP on post-operative pain.

### Strength and limitation

This review was compatible with the framework of the PRISMA-ScR Checklist in identifying the research question and relevant studies, charting the data and handling, and summarizing and reporting the result s[85]. A comprehensive search with no language restrictions was conducted. The screening of records and data extraction were done by two independent reviewers. All variables regarding the impact of PSP time in endodontically treated teeth were considered, which were compatible with bibliometric analysis.

### Implications for further research

Considering controversial outcomes on apical microleakage and scarcity of studies on other variables, further investigations, particularly clinical studies, are suggested. Several materials and techniques were applied, which might have affected the outcomes, indicating a lack of a standard reporting checklist and standard control group.

## Conclusion

The number of studies on the comparison of immediate and delayed PSP increased over time; however, almost all studies were experimental. The studied variables included apical microleakage, coronal microleakage, bacterial infiltration, bond strength of posts, presence of voids, apical displacement of the filling material, adhesion of the remaining filling material, and post-operative pain. Most of the evidence indicated lower apical microleakage and lower residues into the root canal after immediate PSP, lower post-operative pain in delayed PSP, and no difference regarding bacterial microleakage and displacement of filling materials. Considering coronal microleakage, presence of voids, and bond strength, outcomes of the evidence were heterogeneous, indicating that future studies are needed to explore this issue. However, it remains unclear whether factors such as gutta-percha removal methods, obturation techniques, sealer properties, type of cement and adhesive, length of the remaining filling material, and irrigation protocol are effective or not, which requires further investigations.

## Supplementary Information

**Additional file 1.** Online Resource.

## Data Availability

The datasets used and/or analyzed during the current study are available from the corresponding author on reasonable request.

## Abbreviations

PSP: post space preparation
RCT: randomized controlled trial
SEM: scanning electron microscopy
MTA: mineral trioxide aggregate
micro-CT: micro computed tomography
