# Supplementary material for: Immediate and Delayed Post Space Preparations in Endodontically Treated Teeth: A Scoping Review
Source: BMC Oral Health. 2022 Dec 21;22:625. doi: 10.1186/s12903-022-02564-w (PMC9773456; doi:10.1186/s12903-022-02564-w)
Supplement: Supplementary file 1 — Additional file 1. Online Resource. [file 12903_2022_2564_MOESM1_ESM.docx]

**Supplementary Table S1**. Preferred Reporting Items for Systematic reviews and Meta-Analyses extension for Scoping Reviews (PRISMA-ScR) Checklist

| **SECTION** | **ITEM** | **PRISMA-ScR CHECKLIST ITEM** | **REPORTED ON PAGE #** |
| --- | --- | --- | --- |
| **TITLE** | | | |
| Title | 1 | Identify the report as a scoping review. | Page 1 |
| **ABSTRACT** | | | |
| Structured summary | 2 | Provide a structured summary that includes (as applicable): background, objectives, eligibility criteria, sources of evidence, charting methods, results, and conclusions that relate to the review questions and objectives. | Page 1 |
| **INTRODUCTION** | | | |
| Rationale | 3 | Describe the rationale for the review in the context of what is already known. Explain why the review questions/objectives lend themselves to a scoping review approach. | Page 2 |
| Objectives | 4 | Provide an explicit statement of the questions and objectives being addressed with reference to their key elements (e.g., population or participants, concepts, and context) or other relevant key elements used to conceptualize the review questions and/or objectives. | Page 1, paragraph 4 |
| **METHODS** | | | |
| Protocol and registration | 5 | Indicate whether a review protocol exists; state if and where it can be accessed (e.g., a Web address); and if available, provide registration information, including the registration number. | Page 2, paragraph 4 , page 1 |
| Eligibility criteria | 6 | Specify characteristics of the sources of evidence used as eligibility criteria (e.g., years considered, language, and publication status), and provide a rationale. | Page 2, paragraph 5 |
| Information sources* | 7 | Describe all information sources in the search (e.g., databases with dates of coverage and contact with authors to identify additional sources), as well as the date the most recent search was executed. | Page 3, paragraph 2 See the Supplemental Table S2 |
| Search | 8 | Present the full electronic search strategy for at least 1 database, including any limits used, such that it could be repeated. | Page 3, paragraph 2 |
| Selection of sources of evidence† | 9 | State the process for selecting sources of evidence (i.e., screening and eligibility) included in the scoping review. | Page 3, paragraph 3 |
| Data charting process‡ | 10 | Describe the methods of charting data from the included sources of evidence (e.g., calibrated forms or forms that have been tested by the team before their use, and whether data charting was done independently or in duplicate) and any processes for obtaining and confirming data from investigators. | Page 3, paragraph 4 |
| Data items | 11 | List and define all variables for which data were sought and any assumptions and simplifications made. | Page 3, paragraph 4 |
| Critical appraisal of individual sources of evidence§ | 12 | If done, provide a rationale for conducting a critical appraisal of included sources of evidence; describe the methods used and how this information was used in any data synthesis (if appropriate). | n/a |
| Synthesis of results | 13 | Describe the methods of handling and summarizing the data that were charted. | Page 3, paragraph 5 |
| **RESULTS** | | | |
| Selection of sources of evidence | 14 | Give numbers of sources of evidence screened, assessed for eligibility, and included in the review, with reasons for exclusions at each stage, ideally using a flow diagram. | Page 3, paragraph 6, Page 4, paragraph 1 |
| Characteristics of sources of evidence | 15 | For each source of evidence, present characteristics for which data were charted and provide the citations. | Page 5, paragraph 1 |
| Critical appraisal within sources of evidence | 16 | If done, present data on critical appraisal of included sources of evidence (see item 12). | n/a |
| Results of individual sources of evidence | 17 | For each included source of evidence, present the relevant data that were charted that relate to the review questions and objectives. | Page 5 to 21 |
| Synthesis of results | 18 | Summarize and/or present the charting results as they relate to the review questions and objectives. | Table 1-7 |
| **DISCUSSION** | | | |
| Summary of evidence | 19 | Summarize the main results (including an overview of concepts, themes, and types of evidence available), link to the review questions and objectives, and consider the relevance to key groups. | Page 22,23 |
| Limitations | 20 | Discuss the limitations of the scoping review process. | Page 23, paragraph 4 |
| Conclusions | 21 | Provide a general interpretation of the results with respect to the review questions and objectives, as well as potential implications and/or next steps. | Page 23,paragraph 6 |
| **FUNDING** | | | |
| Funding | 22 | Describe sources of funding for the included sources of evidence, as well as sources of funding for the scoping review. Describe the role of the funders of the scoping review. | Page 24 |

JBI = Joanna Briggs Institute; PRISMA-ScR = Preferred Reporting Items for Systematic reviews and Meta-Analyses extension for Scoping Reviews.

* Where *sources of evidence* (see second footnote) are compiled from, such as bibliographic databases, social media platforms, and Web sites.

† A more inclusive/heterogeneous term used to account for the different types of evidence or data sources (e.g., quantitative and/or qualitative research, expert opinion, and policy documents) that may be eligible in a scoping review as opposed to only studies. This is not to be confused with *information sources* (see first footnote).

‡ The frameworks by Arksey and O’Malley (6) and Levac and colleagues (7) and the JBI guidance (4, 5) refer to the process of data extraction in a scoping review as data charting*.*

§The process of systematically examining research evidence to assess its validity, results, and relevance before using it to inform a decision. This term is used for items 12 and 19 instead of "risk of bias" (which is more applicable to systematic reviews of interventions) to include and acknowledge the various sources of evidence that may be used in a scoping review (e.g., quantitative and/or qualitative research, expert opinion, and policy document).

*From:*Tricco AC, Lillie E, Zarin W, O'Brien KK, Colquhoun H, Levac D, et al. PRISMA Extension for Scoping Reviews (PRISMAScR): Checklist and Explanation. Ann Intern Med. 2018;169:467–473. [doi: 10.7326/M18-0850](http://annals.org/aim/fullarticle/2700389/prisma-extension-scoping-reviews-prisma-scr-checklist-explanation).

**Supplementary Table S2**. Search strategy for each database

| **Database** | **Search line** | **Number of retrieved records** |
| --- | --- | --- |
| **PubMed** | ((((post OR dowel OR fiber post OR intracanal post OR intraradicular post) AND (space AND prepar*)) AND (time OR timing OR immediate* OR early OR late OR delay*))) | 412 |
| **Web of Science** | ( ( ( ( ( post OR dowel OR fiber AND post OR intracanal AND post OR intraradicular AND post ) AND ( space AND prepar* ) ) AND ( time OR timing OR immediate* OR early OR late OR delay* ) ) ) ) (Topic) | 588 |
| **Scopus** | TITLE-ABS-KEY ( ( ( ( ( post OR dowel OR fiber AND post OR intracanal AND post OR intraradicular AND post ) AND ( space AND prepar* ) ) AND ( time OR timing OR immediate* OR early OR late OR delay* ) ) ) ) | 911 |
| **Cochrane** | ((((post OR dowel OR fiber post OR intracanal post OR intraradicular post) AND (space AND prepar*)) AND (time OR timing OR immediate* OR early OR late OR delay*))) in Title Abstract Keyword - (Word variations have been searched) | 210 |
| **ProQuest** | pub((((((post OR dowel OR fiber AND post OR intracanal AND post OR intraradicular AND post) AND (space AND prepar*)) AND (time OR timing OR immediate* OR early OR late OR delay*))))) OR ab((((((post OR dowel OR fiber AND post OR intracanal AND post OR intraradicular AND post) AND (space AND prepar*)) AND (time OR timing OR immediate* OR early OR late OR delay*)))))Limits applied: Source type: Dissertations & Theses, Scholarly Journals | 385 |
| **WorldCat** | 'ti:((((post OR dowel OR fiber post OR intracanal post OR intraradicular post) AND (space AND prepar*)) AND (time OR timing OR immediate* OR early OR late OR delay*)))' | 72 |
| **Google Scholar** | "immediate" "delayed" "post space preparation" | 100 |
| **Hand search** | references list of all included studies, two endodontics journals including International Endodontic Journal; Journal of Endodontics, two text books including 11th Cohen's Pathways of the Pulp; 7th Ingle’s Endodontics | 144 |

**Supplemental Table S3.** Excluded records at the full-text assessment phase with reasons.

| **Reasons for exclusion** | **References** |
| --- | --- |
| **Full text of records was not possible to access** | 1. Gerogianni P, Chrepa V, Haney S. CAT OF THE MONTH. Critically Appraised Topics. Time of Post Space Preparation Affects the Amount of Coronal Microleakage in Endodontically Treated Teeth When a Resin Sealer Is Used (UT CAT# 2773). Texas dental journal. 2015;132(5):302.  2. Ibrahim R, Seef R, Seniour S. The effect of immediate and delayed post space preparation and post length on the apical seal. Egyptian dental journal. 1995;41(4):1457-62.  3.Sawsan T AZ, El Sayed M. The effect of immediate post-space preparation on the apical seal of endodontically treated teeth obturated with various root canal sealers. Al-Azhar Dental Journal.1993;8(5):575-85. |
| **Immediate time of post space preparation was considered** | 1. Roitman ML, Pinasco LB, Loiacono R, Panetta VC, Anaise CA, Rodríguez PA. Efficacy of different instruments for the mechanical removal of the smear layer in immediate post preparations: a comparative study. Acta odontologica latinoamericana: AOL. 2021;34(2):166-72. |
| **Delay time of post space preparation was considered** | 1. De Nys M, Martens L, De Coster W, Thys M. Evaluation of dowel space preparation on the apical seal using an image processing system. International Endodontic Journal. 1989;22(5):240-7.  2. Al-Shimmary AH, Al-Huwaizi HF. Comparison of apical seal of four obturation techniques after delayed post space preparation. Journal of baghdad college of dentistry. 2012;24(special issue 2). |

Supplementary Figure S1. Top journals with the highest number of articles published regard PSP.

Supplementary Figure S2. The percentage of country which contributing in publishing regard PSP.


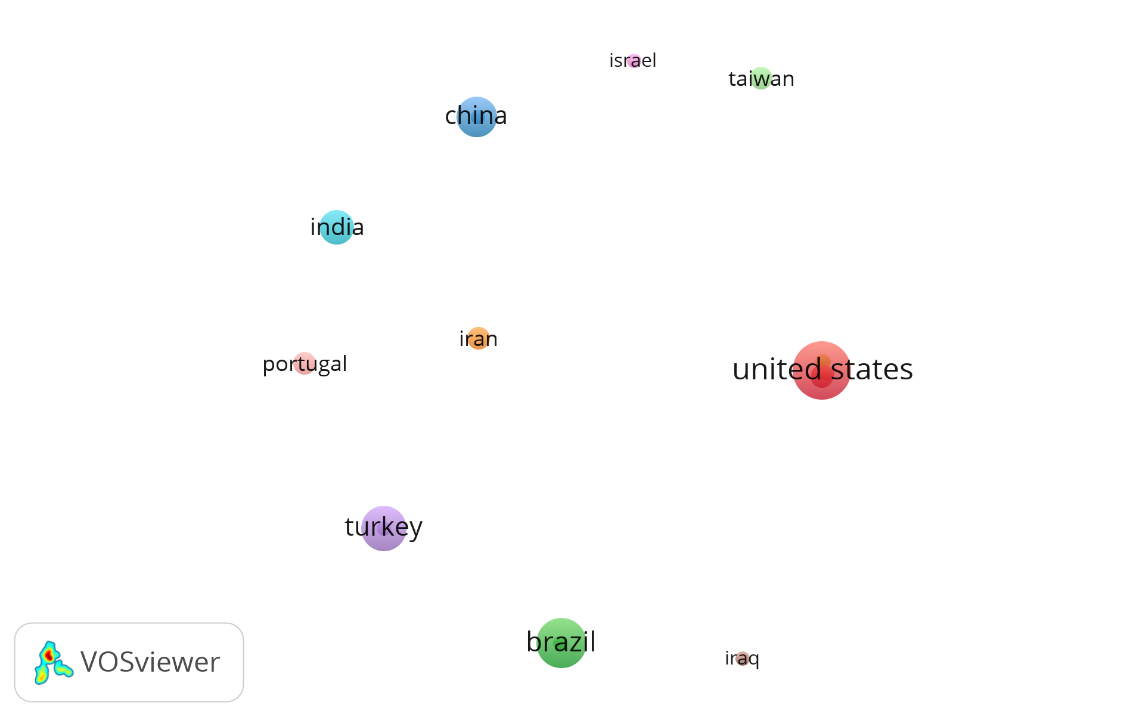


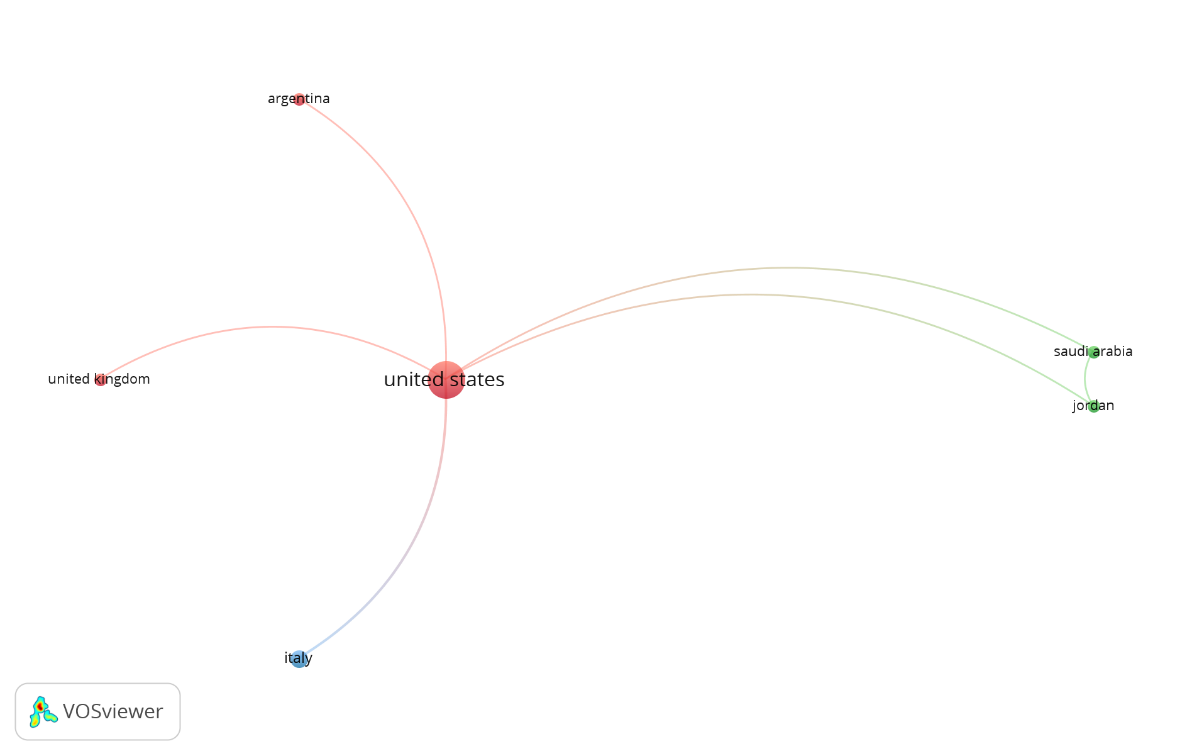


Supplementary Figure S3. The visualization map for origin of countries which contributing in publishing regard PSP (a). Zoomed on highest part of collaboration b).


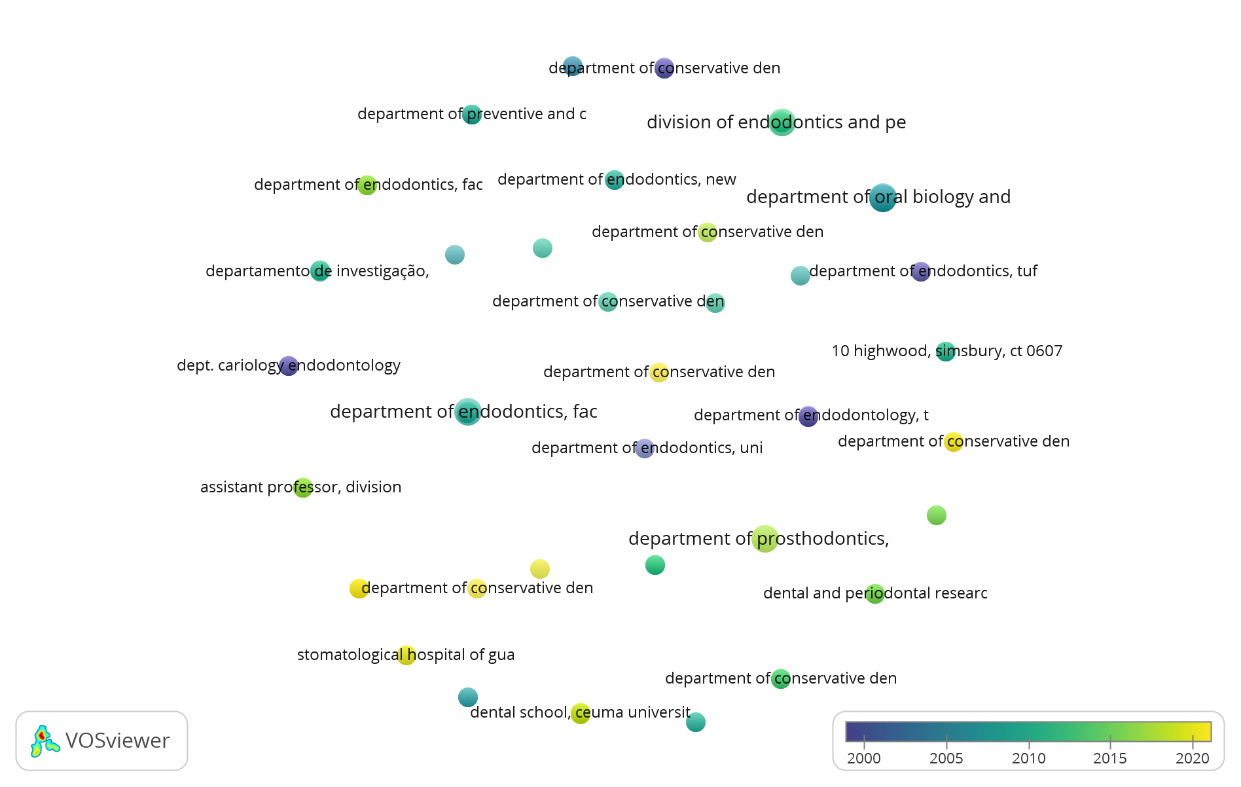


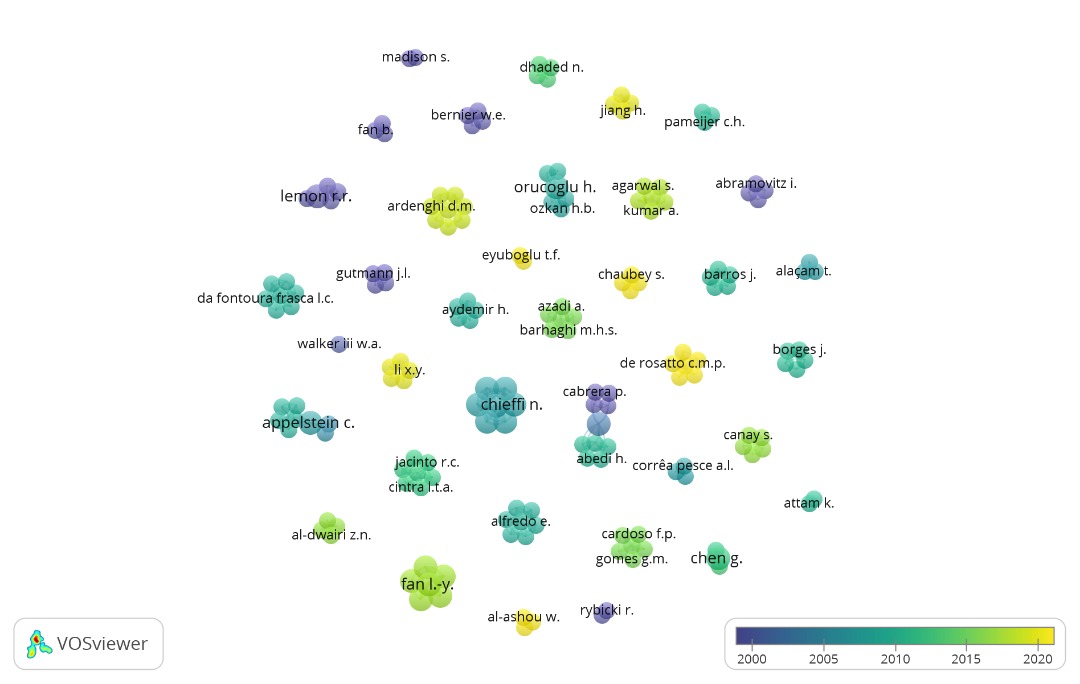


Supplementary Figure S4. Organization (a) and author (b) network visualization of records published regard PSP.
